# Supplementary material for: The interactome of intact mitochondria by cross-linking mass spectrometry provides evidence for coexisting respiratory supercomplexes
Source: Mol Cell Proteomics. 2017 Dec 8;17(2):216–32. doi: 10.1074/mcp.RA117.000470 (PMC5795388; doi:10.1074/mcp.RA117.000470)
Supplement: Supplemental Data [file supp_RA117.000470_133922_0_supp_23978_fzffwf.zip › spectra_annotation/mito_spectra_annotation/347-1-5-1-5-1.pdf]

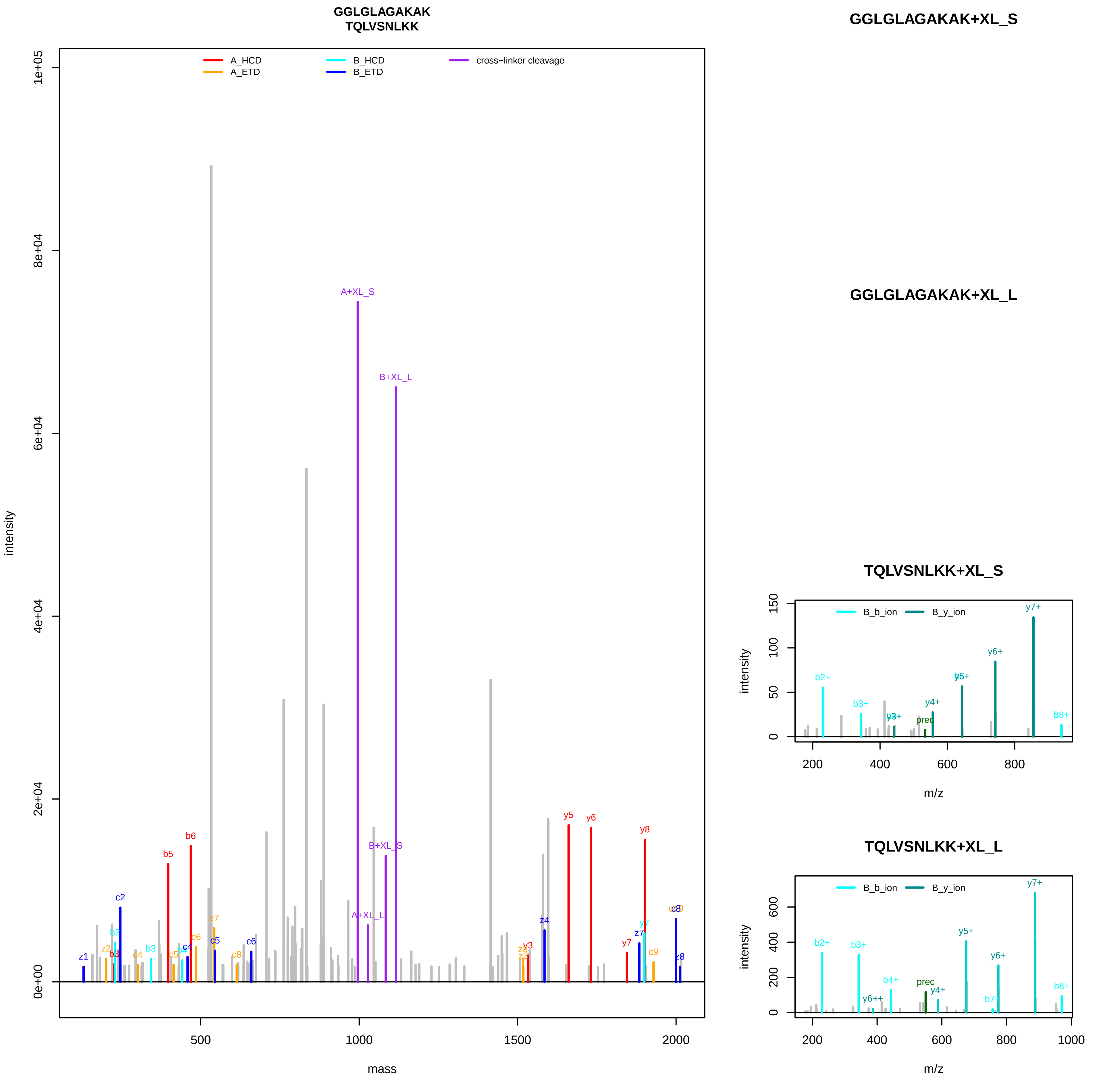
GGLGLAGAKAK
TQLVSNLKK
A_HCD
A_ETD
B_HCD
B_ETD
cross−linker cleavage
intensity
mass
A+XL_S
B+XL_L
B+XL_S
A+XL_L
b5
b6
y5
y6
y7
y8
c2
c5
c6
c7
c8
c9
z1
z4
z7
z8
GGLGLAGAKAK+XL_S
GGLGLAGAKAK+XL_L
TQLVSNLKK+XL_S
TQLVSNLKK+XL_L
B_b_ion
B_y_ion
b2+
b3+
b4+
b7+
b8+
y4+
y5+
y6+
y7+
y6++
prec
m/z
